# Osteogenesis Imperfecta Type 4 With COL1A2 c.1135G>A (p.Gly379Arg) Variant: Unmodified by Concurrent ALPL c.1559delT (p.Leu520ArgfsTer86) Carrier Status: A Case Report

**DOI:** 10.7759/cureus.108052

**Published:** 2026-04-30

**Authors:** Kento Nomura, Yoichiro Oda, Shuhei Yamaguchi, Tomoyuki Ito, Yoshiyuki Namai

**Affiliations:** 1 Department of Pediatrics, Ohta General Hospital Foundation, Ohta Nishinouchi Hospital, Koriyama, JPN; 2 Department of Pediatrics, Chigasaki Municipal Hospital, Chigasaki, JPN

**Keywords:** alpl, col1a2, dominant-negative effect, hypophosphatasia, osteogenesis imperfecta

## Abstract

Osteogenesis imperfecta (OI) is a hereditary connective tissue disorder primarily caused by pathogenic variants in type I collagen genes, while hypophosphatasia (HPP) is a metabolic bone disease resulting from reduced activity of tissue-nonspecific alkaline phosphatase (ALP) due to *ALPL* variants.

We report the case of a male infant initially referred for evaluation of femoral bowing and low serum ALP, suggestive of HPP. Prenatal ultrasonography demonstrated limb shortening, and postnatal imaging confirmed femoral bowing; notably, blue sclerae were absent. Biochemical analysis showed persistently low ALP with mildly elevated urinary phosphoethanolamine. Genetic testing identified a heterozygous *ALPL* variant, c.1559delT (p.Leu520ArgfsTer86), a frequent allele in Japan recognized for its association with an asymptomatic carrier phenotype. Subsequent eruption of the primary tooth revealed dentinogenesis imperfecta. Further genetic evaluation demonstrated a previously reported heterozygous *COL1A2* variant, c.1135G>A (p.Gly379Arg). Based on the clinical constellation of perinatal femoral deformity, dentinogenesis imperfecta, and absent blue sclerae, a diagnosis of OI, moderate form (Sillence type 4), *COL1A2*‐related (OI type 4) was established.

Although prenatal femoral bowing is a common feature of both OI and HPP, the absence of characteristic rachitic features led us to conclude that the bone abnormalities were consistent with a diagnosis of OI type 4. This case suggests that the *ALPL* c.1559delT carrier state may not significantly modify the phenotype of OI.

## Introduction

Osteogenesis imperfecta (OI) is a hereditary connective tissue disorder characterized primarily by bone fragility. Clinical manifestations include recurrent fractures, bone deformities, and short stature, as well as extraskeletal features such as blue sclerae, dentinogenesis imperfecta, and sensorineural hearing loss [1]. The most common causative genes are *COL1A1* and *COL1A2*, which encode the α chains of type I collagen [2]. However, recent advances in genetic research have identified the involvement of numerous genes, leading to the recognition of 37 distinct categories in the *Nosology of Genetic Skeletal Disorders: 2023 Revision* [3].

Hypophosphatasia (HPP) is a metabolic bone disease characterized by reduced activity of tissue-nonspecific alkaline phosphatase (TNSALP) caused by pathogenic variants in the *ALPL* gene. Decreased TNSALP activity results in impaired degradation of pyrophosphate, leading to defective mineralization of bone and teeth [4]. The clinical spectrum of HPP is highly variable, encompassing six phenotypes: perinatal lethal, prenatal benign, infantile, childhood, and adult HPP, as well as odontohypophosphatasia. [5]. Although HPP is generally inherited in an autosomal recessive pattern, some heterozygous variants may exert a dominant-negative effect, leading to milder clinical manifestations [5,6]. With the development of enzyme replacement therapy, early diagnosis has become increasingly important.

This case report presents a patient with OI who also carries a heterozygous *ALPL* variant previously described as an asymptomatic carrier state for HPP.

## Case presentation

**Figure 1:**
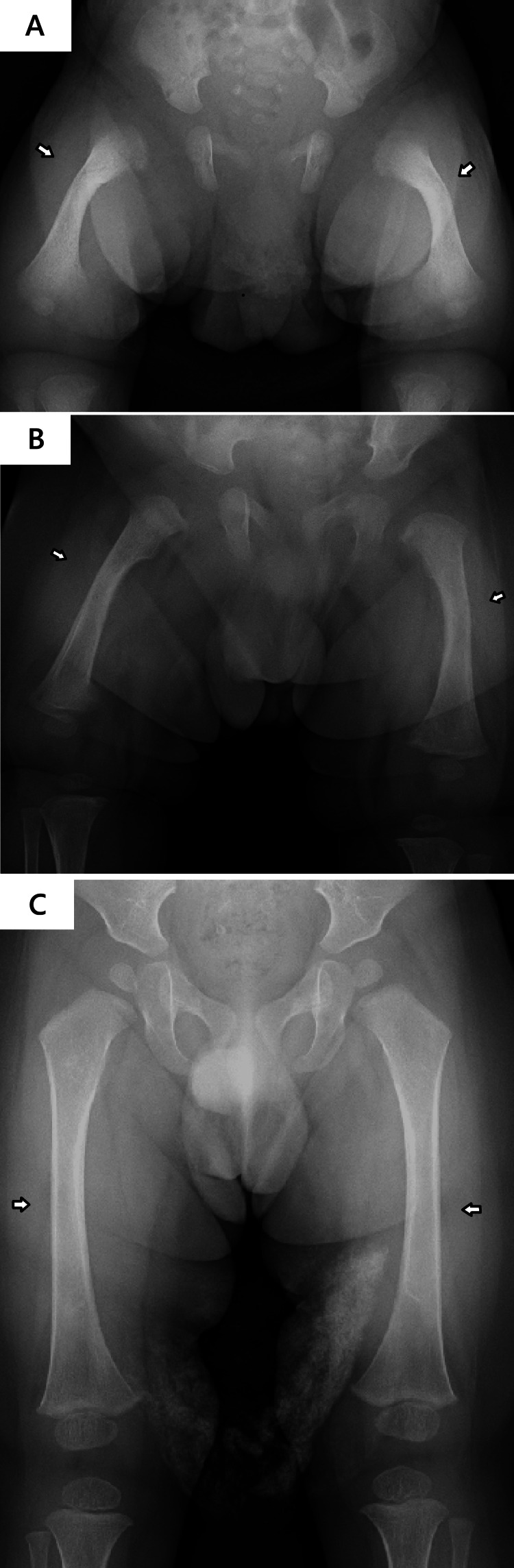
Serial radiographs demonstrating spontaneous resolution of femoral bowing. (A) Birth: Bilateral femoral bowing. Note the absence of rachitic features such as metaphyseal fraying or cupping, which are characteristic of HPP. (B) Two months: Progressive remodeling of femoral bowing. (C) 14 months: Near-normal femoral morphology. HPP: hypophosphatasia.

A two-month-old boy was referred to our hospital for investigation of low serum alkaline phosphatase (ALP) and suspected HPP. Fetal ultrasonography at 33 weeks of gestation had revealed shortening of the limbs. He was born at 38 weeks via spontaneous vaginal delivery, with birth parameters including a length of 47.0 cm (−0.71 SD), weight of 3168 g (0.91 SD), and head circumference of 34.0 cm (0.68 SD). Postnatal radiography demonstrated femoral bowing (Figure 1A).

**Figure 2:**
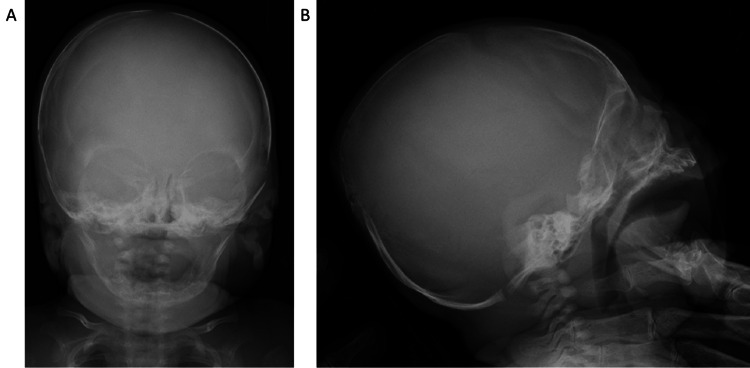
Radiograph of skull at two months of age. (A) Anteroposterior view and (B) lateral view. There is no evidence of Wormian bones.

Upon examination at two months of age, growth parameters were within normal limits (length 58.2 cm (0.15 SD); weight 6345 g (1.15 SD)). The patient exhibited age-appropriate activity with normal muscle tone. Notably, blue sclerae were absent. Radiographs revealed interval improvement in femoral bowing (Figure 1B), while no Wormian bones were observed in the skull (Figure 2). Laboratory evaluation confirmed persistently low serum ALP (130 and 124 U/L on repeated testing; reference range (RR): 171.5-570.5 U/L), as determined by International Federation of Clinical Chemistry and Laboratory Medicine (IFCC) reference method (Table 1). Concomitant biochemical analysis revealed serum calcium at 10.6 mg/dL (RR: 8.5-11.0 mg/dL), phosphate at 5.9 mg/dL (RR: 4.5-8.0 mg/dL), 25-hydroxyvitamin D (25OHD) at 9.6 ng/mL (RR: 20-50 ng/mL), and plasma intact parathyroid hormone (iPTH) at 21 pg/mL (RR: 10-65 pg/mL). Urinary phosphoethanolamine-to-creatinine ratio (PEA/Cr) was minimally elevated at 48.9 mmol/mol Cr (reference range: 0-42 mmol/mol Cr). Family history was significant for the mother (height 162 cm (0.71 SD)), who reported dental complications (enamel delamination and fragile teeth) and two low-energy fractures of the digits, though she lacked blue sclerae. The medical history of the father (height 169 cm (−0.42 SD)) was unremarkable.

**Table 1:** Initial laboratory findings at two months of age. IFCC: International Federation of Clinical Chemistry and Laboratory Medicine reference method; PEA: phosphoethanolamine; PEA/Cr: phosphoethanolamine-to-creatinine ratio.

Parameters	Result	Unit	Reference range
Biochemistry			
Total protein	5.9	g/dL	5.0-6.7
Albumin	4.2	g/dL	3.1-4.5
Total bilirubin	0.71	mg/dL	0.15-1.35
Alkaline phosphatase (IFCC)	130	U/L	70-345
Blood urea nitrogen	4.5	mg/dL	2.5-14.1
Creatinine	0.19	mg/dL	0.14-0.30
Sodium	139	mmol/L	134.9-142.8
Potassium	5.0	mmol/L	4.18-5.72
Chloride	106	mmol/L	101.0-110.7
Calcium	10.6	mg/dL	8.99-11.00
Inorganic phosphorus	5.9	mg/dL	4.60-7.30
Parathyroid hormone	20	pg/mL	15-65
25-hydroxyvitamin D	9.6	ng/mL	20-50
Complete blood cell count			
White blood cell count	12.3	×10^3^/µL	4.60-18.80
Hemoglobin	12.3	g/dL	9.3-13.6
Platelet count	495	×10^3^/µL	260-850
Urine			
Urinary calcium	5.5	mg/dL	
Urinary creatinine	24.48	mg/dL	
Urinary PEA	105.7	μmol/L	
Urinary PEA/Cr ratio	48.9	mmol/mol Cr	0-42

**Figure 3:**
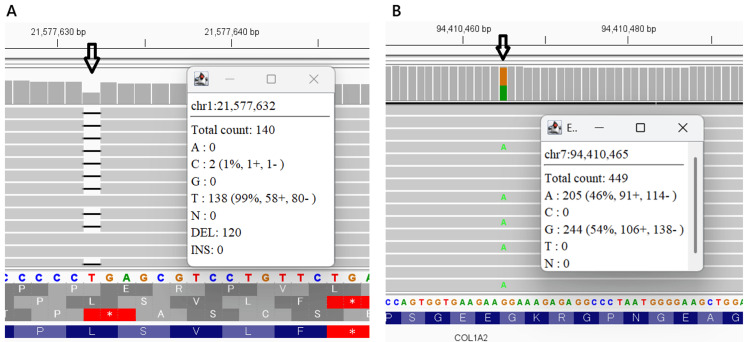
Integrative Genomics Viewer display of targeted next-generation sequencing. (A) Detection of a heterozygous frameshift variant in *ALPL*, c.1559delT (p.Leu520ArgfsTer86). (B) Detection of a heterozygous missense variant in *COL1A*2, c.1135G>A (p.Gly379Arg).

**Figure 4:**
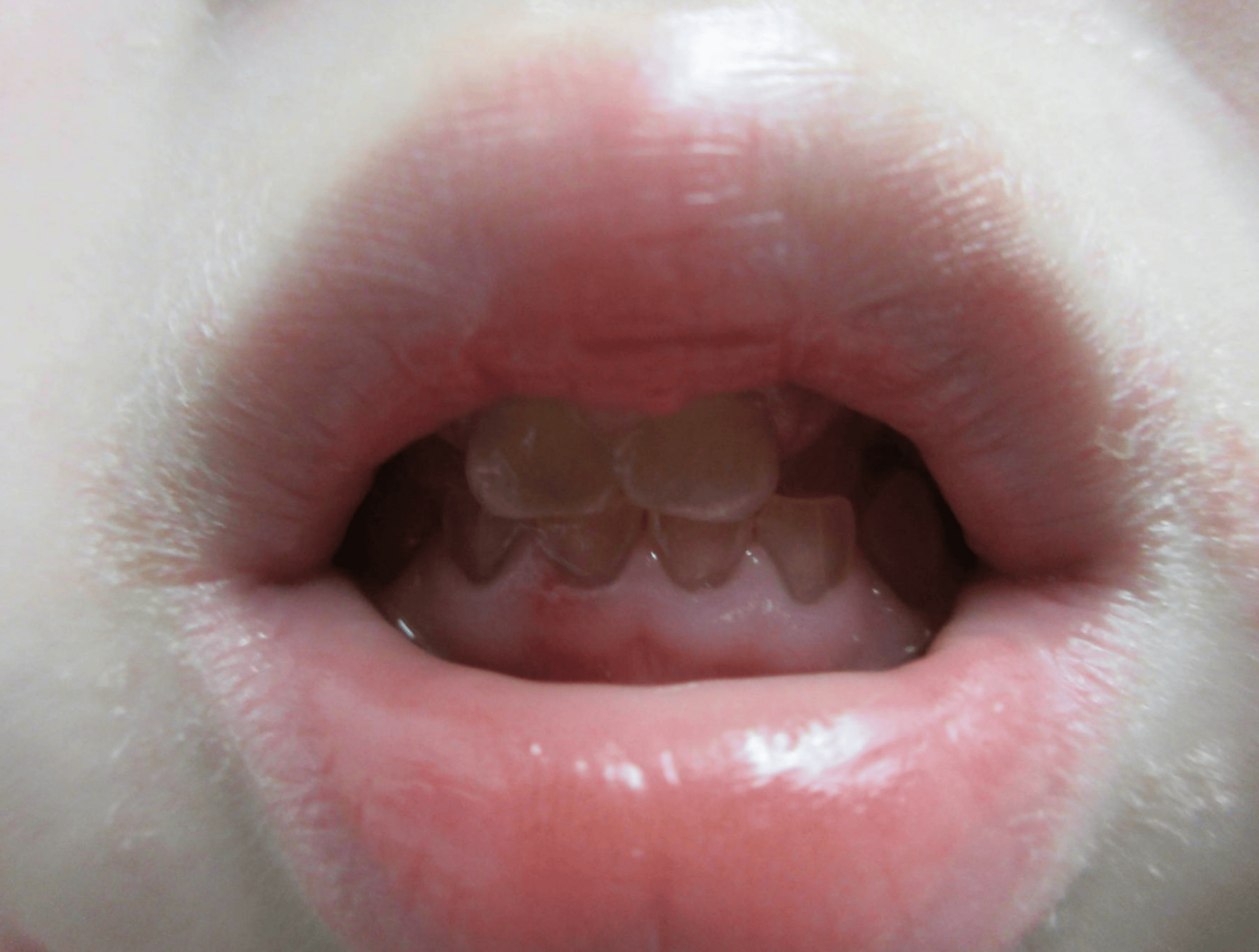
Oral manifestation of the patient at three years. The primary dentition at three years of age, exhibiting characteristic opalescent discoloration and enamel attrition indicative of dentinogenesis imperfecta.

Targeted next-generation sequencing for *ALPL*, performed at the Kazusa DNA Research Institute (Chiba, Japan) initially identified a heterozygous *ALPL* variant, c.1559delT (p.Leu520ArgfsTer86) (Figure 3A). This specific variant is highly prevalent in Japanese population and reported to lead to asymptomatic carrier state [7-9]. However, at 11 months of age, eruption of primary tooth revealed dentinogenesis imperfecta (Figure 4). Targeted next-generation sequencing for bone fragility-related genes (including *BMP1*, *COL1A1*, *COL1A2*, *CRTAP*, *FKBP10*, *IFITM5*, *P3H1*, *PPIB*, and *SERPINF1*), performed at the same institute (Kazusa DNA Research Institute, Chiba, Japan), identified a heterozygous *COL1A2* variant, c.1135G>A (p.Gly379Arg) (Figure 3B). Based on the clinical constellation of perinatal femoral deformity, dentinogenesis imperfecta, and absent blue sclerae, a diagnosis of OI, moderate form (Sillence type 4), *COL1A2*‐related (OI type 4) was established.

**Figure 5:**
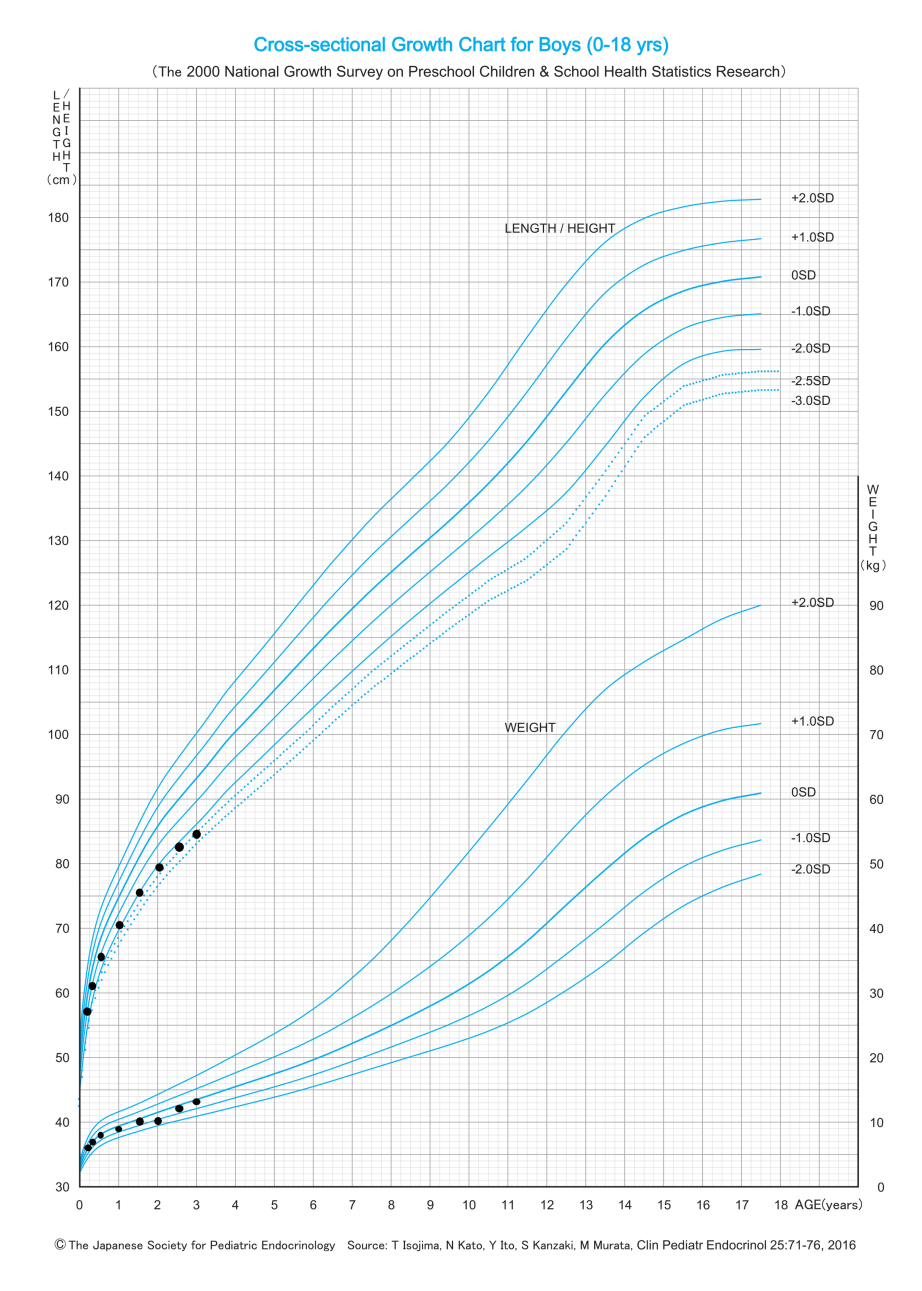
Growth chart of the patient. The development of short stature.

**Figure 6:**
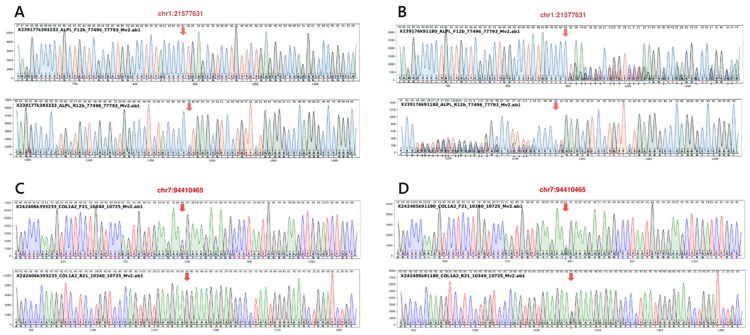
Sanger sequencing of the parents. (A) Wild-type sequence at the *ALPL* c.1559 position in the father. (B) Confirmation of the heterozygous *ALPL* c.1559delT variant in the mother. (C) Wild-type sequence at the *COL1A2* c.1135 position in the father. (D) Confirmation of the heterozygous *COL1A2* c.1135G>A variant in the mother.

Follow-up at 14 months of age showed normalized femoral morphology (Figure 1C). Longitudinal monitoring at 23 months of age revealed that the urinary PEA/Cr had increased to 83.5 mmol/mol Cr (reference range: 0-23 mmol/mol Cr). It is noteworthy that, as reported by Imbard et al., reference ranges for the urinary PEA/Cr ratio exhibit significant age-dependent variations throughout infancy and early childhood [10]. By three years of age, the patient exhibited short stature (84.7 cm (−2.35 SD)) (Figure 5) with normal neurodevelopment, despite resolution of femoral bowing. Notably, no fractures or premature loss of primary teeth occurred. Parental Sanger sequencing confirmed that the symptomatic mother shared both heterozygous *ALPL* and *COL1A2* variants, whereas the father carried neither (Figure 6).

## Discussion

In the present case, both the patient and his mother harbored concurrent variants in the *COL1A2* and *ALPL* genes. The *COL1A2* c.1135G>A (p.Gly379Arg) heterozygous variant has previously been reported in patients with OI type 3 and 4 [11-13]. While Bardai et al. [11] and Holtz et al. [12] classified their respective cases as having OI type 3. Sałacińska et al. reported a case with OI type 4 [13]. Furthermore, Wang et al. described the clinical manifestations as “typical clinical OI features” [14]. In our case, the patient presented with transient perinatal femoral bowing and dentinogenesis imperfecta without blue sclerae, which was consistent with OI type 4. The maternal diagnosis was likewise established as OI type 4 based on the shared genotype and clinical features.

To date, there have been two reports of concurrent heterozygous variants in* COL1A1/2* and *ALPL* associated with marked mineralization defects; notably, the *ALPL* variants in those cases (p.Ser445Pro, p.Glu476Lys) were documented to exert a dominant-negative effect and potentially manifest as the adult form of HPP [15,16]. In contrast, the variant *ALPL *c.1559delT (p.Leu520ArgfsTer86)-the most prevalent in the Japanese population-lacks a dominant-negative effect [7-9,17]. It has been suggested that the TNSALP p.Leu520ArgfsTer86 variant protein undergoes homo-aggregation through disulfide bonding involving the novel residues from frameshift rather than interacting with the wild-type TNSALP [17]. Furthermore, the frameshift results in the loss of the C-terminal glycosylphosphatidylinositol anchor signal sequence [17]. Consequently, despite retaining enzymatic activity, the variant protein fails to be anchored to the cell surface and is hardly secreted into the extracellular space, rendering it unable to perform its physiological functions. These findings suggest that the loss of functional TNSALP is attributable to haploinsufficiency rather than a dominant-negative effect; this likely accounts for why *ALPL* c.1559delT (p.Leu520ArgfsTer86) carriers remain asymptomatic. To the best of our knowledge, there are no reports of bone mineral loss in carriers of the *ALPL* c.1559delT (p.Leu520ArgfsTer86) variant. In these carriers, it has been reported that serum ALP levels are low-normal to slightly low, and PEA/Cr ratios are normal to slightly high, which is consistent with the values observed in our case [7,8].

Previous studies have demonstrated that low vitamin D levels alone are not invariably associated with secondary hyperparathyroidism or the clinical manifestation of rickets [18]. Despite subnormal 25OHD, iPTH remained within reference range, suggesting that decreased vitamin D did not adversely affect bone mineralization. Prenatal femoral bowing is a common feature of both OI and HPP. However, as the characteristic rachitic features of HPP were absent in our patient, the bone abnormalities were deemed consistent with a diagnosis of OI type 4.

## Conclusions

We reported a case with concurrent heterozygous variants in *COL1A2* and *ALPL*. Our findings suggest that the HPP carrier state associated with the *ALPL* c.1559delT variant did not modify the clinical course of OI type 4. Further accumulation of cases is warranted to confirm our findings.
